# *IL23R* and *IL12B* SNPs and Haplotypes Strongly Associate with Crohn's Disease Risk in a New Zealand Population

**DOI:** 10.1155/2010/539461

**Published:** 2010-12-27

**Authors:** Lynnette R. Ferguson, Dug Yeo Han, Alan G. Fraser, Claudia Huebner, Wen Jiun Lam, Angharad R. Morgan

**Affiliations:** ^1^Discipline of Nutrition, Faculty of Medical and Health Sciences, The University of Auckland, Auckland, 1142, New Zealand; ^2^Nutrigenomics, New Zealand; ^3^Department of Medicine, Faculty of Medical and Health Sciences, The University of Auckland, Auckland, 1142, New Zealand

## Abstract

DNA samples from 339 Crohn's disease (CD) and 407 randomly selected controls from the Auckland (New Zealand) IBD project, were genotyped for five common single nucleotide polymorphisms in *IL-23R* (rs11805303, rs7517847, rs1343151, rs11209026, and rs10889677) and two in *IL-12B* (rs1363670 and rs6887695). While the *IL-12B* variants did not show an overall association and other *IL23R* variants led to minor changes in the risk of CD, rs1343151 and/or rs7517847 variants in the *IL-23R* gene strongly reduced the risk of developing CD at both allelic and genotype levels. A significantly decreased risk of first diagnosis of childhood CD was observed in individuals carrying the A allele of rs1343151, or between 17–40 y in individuals carrying the G allele in rs7517847 of *IL-23R*. A significantly decreased risk of ileocolonic or structuring disease was observed in individuals carrying the A allele in either rs11209026 or rs1343151, or the G allele in rs7517847 of *IL-23R*, and when such individuals did develop the disease, they were unlikely to require a bowel resection. Certain haplotypes very strongly modified risk. There was evidence for interactions of *IL-23R* variants with the *NOD2* wild-type (d/d) genotype. Down-regulating the function of the *IL-23R* gene may decrease CD risk in the normal population.

## 1. Introduction

The inflammatory bowel diseases, Crohn's disease (CD) and ulcerative colitis (UC), are common gastrointestinal disorders in various countries, including New Zealand and parts of Europe. In New Zealand, it appears that their incidence is rising, and a geographically-based study in the Canterbury region in 2005 identified close to 1 in 250 people with the disease [1]. CD is also increasing in New Zealand paediatric patients [2]. Although mortality is low, the symptoms can be debilitating, including abdominal cramping and bloody diarrhoea. An increasing number of studies are associating genes with susceptibility to the disease. However, not all identified variants and genes show comparable risks in different countries [3].

Genome-wide association studies (GWAS) are rapidly enhancing our understanding of key genetic polymorphisms in CD, as in many other autoimmune disorders. Indeed, the initial study on *IL23R* helped to provide proof of the power of this technology to detect non-hypothesis based disease associations [4]. Duerr and coworkers [5] utilised a well-phenotyped population, by focussing on a single disease type—ileal CD. As well as associations with two nucleotide oligomerisation domain 2 (*NOD2*; alternatively known as caspase activating recruitment domain or *CARD15*) variants previously implicated in the disease, they also found a strong disease association with chromosomal region 1p31, which had not previously been implicated in CD. The signal associated a single-nucleotide polymorphism (SNP) of the gene encoding the interleukin-23 receptor (*IL-23R*), not with increased disease risk but with decreased risk for CD. The SNP, rs11209026, encodes an amino acid change (Arg381Gln) in the protein product and has functional consequences. Other analyses in two independent cohorts replicated this result, as well as uncovering disease relationships with eight other related noncoding variants within *IL-23R *[6, 7]. Interestingly, some of the closely located SNPs led to protection while others were associated with an increased risk of CD. Duerr and coworkers [5] also investigated SNPs in the adjacent intergenic region containing the *IL*-12 receptor, *b*-2 (*IL-12RB2*) gene, since this is mechanistically associated. However, this gene did not show the same strong associations as those seen for *IL-23R*.

A previous study based in Canterbury, New Zealand, associated* IL-23R* Arg381Gln with CD and possibly also UC in that region of this country [8]. However, the authors reported no significant association between *IL-23R* genotype and IBD phenotypes and also that the association was only seen in those subjects who did not carry variant alleles in *NOD2*. They did not consider other SNPs or other potential interactions.

*IL*-23 is a cytokine that acts as a proinflammatory mediator of autoimmune and chronic inflammatory diseases. In association with *IL*-12, it is part of the T-helper 17 cell axis [9]. The *IL-12B *gene, alternatively known as natural killer cell stimulatory factor 2, cytotoxic lymphocyte maturation factor 2, or p40, encodes the p40 subunit of IL-12B (ligand) and IL-23R (receptor), both of which are dimeric proteins [9]. The *IL-12B* gene contains two major sites, both of which appear susceptible to variation and at which different alleles are associated with variable levels of gene expression. van de Vosse and Ottenhoff [10] reported that mutations in *IL-12B* impair IL-12 and IL-23 responses and predispose subjects to infections caused by mycobacteria and salmonella. Although the original study by Duerr and coworkers [5] did not associate variants in *IL-12B* with effects in *IL-23R*, other studies have done so [11, 12]. 

We have substantially expanded the available data on the New Zealand situation, by considering a wider range of variants in both *IL-12B* and *IL-23R* in our North Island CD population, and by analysing associations with phenotype. We have also done haplotype analyses and considered the possibility of interactions of risk between SNPs in these genes, and in *NOD2*.

## 2. Materials and Methods

### 2.1. Study Participants

The Auckland IBD Project is a population-based study of genetic and environmental determinants of CD aetiology. Patients were recruited between May 2005 and April 2009 through local doctors and surgeries in Auckland, New Zealand, and also other North Island centres. They also responded to media campaigns, for example, local newspaper and television. Controls were also recruited in a similar fashion and included nonaffected spouses of the CD subjects. Participants consented to collection of peripheral blood for DNA extraction and genotyping, and questionnaires were taken away for completion. Data were scrutinised for accuracy and completion and subjects recontacted for clarification where necessary. 

A total of 746 subjects (339 CD patients and 407 controls) subjects consented to take part. The cases in this study are a random subset of the Caucasian participants of the Auckland IBD Project. CD was defined using standard diagnostic criteria [13]. Cases were phenotyped according to the Montreal Classification systems, allowing genotype-phenotype analysis to be performed. CD diagnosis was confirmed in each patient by review of his or her case notes. Patients were excluded from the cohort if there was insufficient information to confirm a diagnosis of CD. All participants self-reported European ancestry, and patients who self-reported having any Maori or other non-Caucasian ancestry were not included in the dataset. Clinical and demographic characteristics of the Caucasian CD cohort for this study are given in Table 1. 

The study was conducted under ethical protocol MEC/04/12/011, authorised through the New Zealand Multi-Region Human Ethics Committee. All study subjects gave informed consent. DNA was extracted from the blood samples using Qiagen's DNA extraction kit and following the manufacturer's instructions.

### 2.2. Genotyping

Genotyping for the polymorphisms in the *IL-12B* gene (GenBank: NM_002187, g.158716689C > G, rs1363670 and g.158755223G > C, rs6887695) and *IL-23R* gene (GenBank: NM_144701, g.44034C > T, rs11805303; g.50187T > G, rs7517847; g.87647G > A, rs1343151; c.1142G > A, rs11209026 and c*2370C > A, rs10889677) used the MassARRAY and iPlex systems of the Sequenom genotyping platform (Sequenom, San Diego, CA), which uses the MALDI-TOF primer extension assay [14–16], according to the manufacturers' recommendations. However, this method failed for rs11209026, which was genotyped using the ABI TaqMan MGB diallelic discrimination system. A custom-made, quality-controlled and functionally tested genotyping assay (Assay-by-Design online service) was obtained from Applied Biosystems (Melbourne, Australia).

All sample plates contained cases, controls, blanks, and duplicate samples. Quality control measures including independent double genotyping, blind to sample identity and blind to the other caller and duplicates, were checked to ensure that there were no discrepancies.

### 2.3. Statistical Analysis

The allelic trend test [17] and Fisher's exact genotypic test were used to compare frequencies between case and control allele. An exact test was used to test for departures from Hardy-Weinberg equilibrium (HWE) in the case and the control samples [18]. Allelic odds ratios and confidence intervals for the allelic odds ratios were calculated under the assumption of HWE in the cases and the control groups. We also used a Cochran-Armitage Trend test to consider allelic differences. A multiple testing correction on phenotype analyses was conducted using the false discovery rate (FDR). Linkage disequilibrium (LD) for *IL12B* and *IL23R* for CEU population was evaluated using Haploview 4.2, and the block was defined by the Four Gamete Rule (http://www.broadinstitute.org/haploview). Haplotype analyses for *IL12B* (rs1363670 and rs6887695) and* IL23R* (rs10889677, rs11805303, rs1343151, rs11209026, and rs7517847) were carried out using haplo.stats package in R [19] to test for association of these haplotypes with CD. Haplotype frequencies were estimated, and association analyses were performed with respect to CD in patients and controls. A score for each haplotype (Hap-score) was calculated. We also performed an exploratory analysis of allele frequency differences between controls and patient subgroups defined using the clinical characteristics. These analyses were carried out using R and SAS (V9.1 SAS Institute., Cary, NC, USA).

The statistical interaction for CD-associated variants in *NOD2* with *IL-12B* and *IL-23R* was also investigated. The three *NOD2* SNPs (rs2066844, rs2066845, and rs2066847) were classified as homozygous wild-type (d/d), heterozygous carrier (d/D), or homozygous mutant carrier including compound heterozygotes (D/D), as previously identified in [20].

## 3. Results

### 3.1. Data Quality

Each of the 7 SNPs had a genotyping call rate greater than 90% and was in HWE in the controls. Although *IL23R* (rs1343151 and rs7517847) was out of HWE in the CD cases, it demonstrated highly significant associations with IBD.

### 3.2. Characteristics of Case and Control Populations

Of 339 cases, 88 (28.3%) cases have other families and relatives with IBD, 99 (31.8%) had bowel resections, 112 (46.7%) smoked at diagnosis, and 44 (14.2%) showed extra intestinal manifestations of CD (Table 1). The numbers of cases successfully genotyped did not differ from the number of controls (data not shown).

### 3.3. Allelic and Genotype Differences

There were no overall differences in allelic or genotype frequencies between cases and controls for either of the variant alleles in *IL-12B* (Table 2). However, there were significant differences (*P* < .05) between CD patients and controls in the allele frequency for four *IL23R* SNPs (rs11209026, rs11805303, rs1343151, and rs7517847) and in genotypic frequency for three *IL23R* SNPs (rs10889677, rs1343151, and rs7517847).

For the rs10889677 SNP, the heterozygous A/C genotype showed a significantly increased risk of CD as compared with the homozygous C/C genotype. For the rs11209026 SNP, a significant decrease in the frequency of the A allele was observed in CD patients. For the rs11805303 SNP, a significant increase in the frequency of the T allele was observed in CD. For the rs1343151 polymorphism, a significant decrease in the frequency of the A allele was observed in CD patients. A strongly decreased risk of CD was observed in A/A homozygous individuals as compared with G/G homozygous individuals. Considering the rs7517847 polymorphism, a significantly decreased frequency of the G allele was observed in CD patients. A significantly decreased risk of CD was observed in G/G homozygote as compared with T/T homozygous individuals.

### 3.4. Phenotype Analyses

Phenotype analyses for *IL-12B* are shown in Table 3(a), and for *IL-23R* in Table 3(b). A significantly decreased risk of CD was observed in individuals carrying the A allele of rs1343151 in *IL-23R* in both females and males or carrying the G allele in rs7517847 of *IL-23R* in males.

A significantly decreased risk of first diagnosis of CD under 17 years was observed in individuals carrying the A allele of rs1343151 in *IL-23R*. A significantly decreased risk of age at first diagnosis of CD between 17 and 40 years was observed in individuals carrying the A allele of rs1343151 or the G allele in rs7517847 of *IL-23R*.

A significantly decreased risk of ileal disease was observed in individuals carrying the C allele in rs6887695 of *IL-12B*. Similarly, three of the variants of the *IL-23R* gene were associated with having an increased risk of ileal disease. An increased risk of ileal disease was evident for those carrying the T allele in rs11805303 whilst a decreased risk of ileal disease was associated with the A allele in rs1343151 and the G allele in rs7517847 of *IL-23R*. A significantly decreased risk of ileocolonic disease was observed in individuals carrying the A allele in rs11209026 and rs1343151 or the G allele in rs7517847 of *IL-23R*.

A significantly increased risk of stricturing disease was observed in individuals carrying the C allele in rs1363670 of *IL-12B*. A significantly decreased risk of inflammatory disease was observed for those individuals carrying the A allele in rs1343151 of *IL-23R* a significantly decreased risk of penetrating disease was also observed with this same SNP; and a significantly decreased risk of stricturing disease was also observed with this same SNP. A significantly increased risk of stricturing disease was observed in individuals who have the T allele in rs11805303; a significantly decreased risk of stricturing disease was observed in individuals who have the A allele in rs11209026 and rs1343151 and individuals who have the G allele in rs7517847 of *IL-23R*.

A significantly increased risk of stricturing disease with ileal involvement was observed in individuals who have the C allele in rs1363670 of *IL-12B* and a significantly decreased risk of stricturing disease with ileal involvement in individuals carrying the C allele in rs6887695 of *IL-12B*. A significantly decreased risk of stricturing disease with ileal involvement was seen in individuals carrying the G allele in rs7517847 of *IL-23R*. 

A significantly decreased risk of having any other families and relatives with IBD was observed in individuals carrying the A allele in rs1343151 and the G allele in rs7517847 of *IL-23R*. 

A significantly decreased risk of CD requiring a bowel resection was observed in individuals carrying the A allele in rs11209026 and rs1343151 and the G allele in rs7517847 of *IL-23R*. A significant decrease in the risk of extra intestinal manifestations (EIM) of CD was observed in individuals carrying the A allele in rs10889677 and rs1343151. 

The phenotypes which were significantly associated with both SNPs rs1343151 and rs7517847 of *IL-23R* remain significant after a multiple testing correction applied using the FDR.

None of SNPs in this study was significantly associated with smoking at diagnosis.

### 3.5. Perianal Disease

A total of 42 (13.5%) CD patients had perianal disease. For the rs10889677 SNP, the homozygous A/A and the heterozygous A/C genotype showed a significantly increased risk of perianal disease as compared with the homozygous C/C genotype. A significant increase in the frequency of the A allele rs10889677 was observed in perianal patients. 

For the rs1343151 SNP, the heterozygous A/G genotype showed a significantly decreased risk of perianal disease as compared with the homozygous G/G genotype. A significant decrease in the frequency of the A allele rs1343151 was observed in perianal patients.

### 3.6. Haplotype Analysis

**Figure 1:**
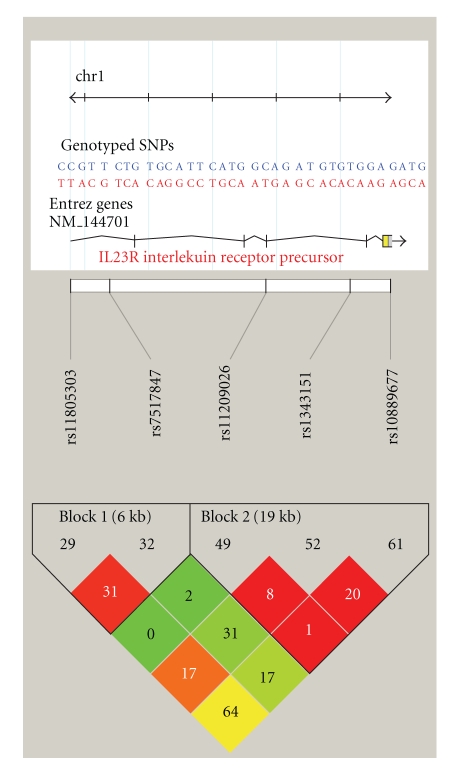
IL23R LD plot.

Two SNPs of *IL-12B* and five SNPs of *IL-23R* disequilibrium block were evaluated for haplotype blocks using the four gamete rule in Haploview. Two haplotype blocks were constructed for *IL-23R* (Figure 1), and Tables 4(a) and 4(b) summarise these results. The block 1 haplotype consisted of two SNPs (rs11805303 and rs7517847). The hap-frequency of haplotype CG was 0.450 in control group, which is significantly higher than that in CD cases (0.358, *P* = .0005). The hap-frequency of haplotype TT was 0.359 in CD cases, which is significantly higher than that in control group (0.305, *P* = .025). Block 2 haplotype consisted of three SNPs (rs10889677, rs11209026, and rs1343151). The hap-frequency of haplotype CGA was 0.296 in the control group, which is significantly higher than that in CD cases (0.232, *P* = .007). The hap-frequency of haplotype AGG was marginally significantly higher in CD cases (0.374) compared with that in control group (0.323, *P* = .056) and CAA was marginally significantly higher in the control group (0.069) than in CD cases (0.044, *P* = .058). No *IL-12B* haplotype was significantly associated with CD (*P* = .148).

### 3.7. Interaction between IL-23R and NOD2 with respect to Crohn's Disease

The statistical interaction for CD-associated variants in *NOD2 *with *IL-12B* and *IL-23R* was investigated. The frequencies and odds ratios of *NOD2* risk genotypes stratified by *IL-23R* (rs10889677, rs11209026, rs11805303, rs1343151, and 7517847) are shown in Table 5, while Table 6 shows frequencies of genotypes of the three *NOD2* SNPs. A statistical interaction was detected between rs1343151 and *NOD2*, whereby carrying the genotype A/A significantly decreased the risk of CD on the background of *NOD2 *genotype d/d. For rs7517847, the genotype G/G showed significantly decreased risk of CD in those individuals with the *NOD2* genotype d/d. No significant interactions were found between rs10889677, rs11209026, rs11805303, or *IL-12B* and *NOD2* genotype. The odds ratios on the background of *NOD2* genotype D/D were not calculated, due to small numbers of D/D cases and controls.

## 4. Discussion

This study confirms the very strong involvement of *IL-23R* and, to a lesser extent, *IL-12B*, in Crohn's disease in New Zealand. It is of interest that Duerr and coworkers [5] utilised a CD population of only a single phenotype—those with ileal involvement—in their original study which uncovered the involvement of *IL-23R* in CD risk. Our own data confirms a relationship with ileal involvement for several of the SNPs studied. The association of this particular variant SNP, rs11209026 (R381Q), in this gene with decreased risk of CD has been confirmed in paediatric populations [6, 7] and also in the large UK database studied by the Wellcome Trust Case Control Consortium [21]. Positive results of varying quality and significance have also been reported from other populations, including Scotland, Continental Europe, North America, New Zealand, Brazil, and Israel [8, 22–27]. However, no associations were seen in a Japanese cohort [28]. The majority of these studies have focussed on the R381Q allele. In our own studies, data for this allele provide nowhere near the statistical significance of other allelic variants in this gene. Our study suggests that rs11209026 may be less important than other markers and also confirms that even closely related SNPs may have opposite effects on CD risk. Several of them are associated with paediatric disease.

Considering *IL-12B*, carrying the rs1363670 C variant increases CD risk while carrying the rs6887695 C variant decreases CD risk. In regard to the *IL-23R* gene, the rs10889677 A variant and the rs11805303 T variant both increase disease risk while both the rs1343151 variant A and the rs7517847 G variant reduce disease risk. That is, some of these closely located SNPs led to protection while others were associated with an increased risk of CD. Similar results were originally published by Duerr et al. [5], who suggested that these apparently contradictory results might be explained by alternative splicing of *IL-23R*. 

For several other alleles in both *IL12B* and *IL-23R*, our own data have confirmed the observations of strong ileal involvement [5]. In particular, a highly significant increased risk of ileal disease was observed in individuals carrying the C allele in rs6887695 of* IL-12B*. Similarly, one of the variants in the *IL-23R* gene decreased the risk of ileal disease (the T allele in rs11805303) while two increased the risk of ileal disease (the A allele of rs1343151 or the G allele of rs7517847). Even stronger statistical significance was observed for ileo-colonic disease in individuals carrying the A allele of rs1343151 or the G allele in rs7517847 of *IL-23R*. Although the risk of inflammatory disease with colonic involvement was not significantly associated with any of the variant SNPs in this study, there were strong associations with stricturing disease for most of the studied SNPs. It is also important that carrying either of two of the *IL-23R* SNPs significantly increased the probability that a bowel resection will be required.

Early studies on CD had considered* IL-12B *as a candidate gene for CD susceptibility, partly because *IL-12* is upregulated in active disease [29–31]. Interleukin-12 is a dimeric protein, consisting of two subunits, p35 and p40, the latter of which is encoded by the *IL-12B* gene. In an animal model of CD, it proved possible to suppress established chronic intestinal inflammation by the use of antibodies to the p40 subunit [32]. It should be noted, however, that antibodies to p40 also suppress the activity of *IL-23*, since these two cytokines have the p40 subunit in common. The most likely interpretation of the accumulating set of data is that *IL-23* plays a more important role than *IL-12 *in chronic inflammation. *IL-23* activates a subset of T cells (TH17 T-cells) leading to the production of the cytokine *IL-17*, which modulates chronic inflammatory diseases. Kobayashi et al. [33] provide evidence that *IL-23* differentially regulates the Th1/Th17 balance in inflammatory bowel diseases.

The present study adds to an increasing number of reports of *IL-23R* importance in susceptibility to autoimmune diseases. Several reports have demonstrated significant associations between carriage of variants in the *IL-23R* with psoriasis vulgaris, psoriatic arthritis, autoimmune thyroid disease, and Graves' ophthalmopathy, but not with arthritis per se, or myocardial infarct [34–37]. It is of some interest that we have found associations with extra intestinal manifestations of disease in the present study.

The functionality of several of the SNPs studied here is currently unknown. Considering possible effects of the SNPs studied in *IL-23R*, rs10889677 is located in the 3′ untranslated region and the variant may lead to overexpression of the receptor. Similarly, each of the other three SNPs is in an intragenic region and may also affect receptor expression. Receptor overexpression would enhance the release of *IL-17*, because it would enhance the differentiation of Th1 helper T cells towards a Th17 subpopulation. Flow-on effects would lead to the release of other cytokines such as TNF-alpha, thereby enhancing chronic inflammation. The SNPs in *IL-12B* may be in linkage disequilibrium with a functional variant. It will be important that future studies begin with more specific information on functionality of the SNPs, preferably based on effects from human studies rather than tissue culture models. 

The present study supports other observations that reducing the expression of *IL-23R* may provide an important therapeutic target, not only for CD but also for other diseases. It was shown that blocking the activity of either *IL-23* itself or its downstream factors *IL-17* and *IL-6* significantly impeded the development of disease in animal models of IBD and multiple sclerosis [38, 39]. More specifically, Becker and coworkers [40] showed that blocking the p40 subunit of either *IL-12* or *IL-23*, but not blocking the p19 unit, was effective in reducing disease symptoms in chemically induced colitis models. The same results were not seen after blocking the *IL-12*/interferon-gamma pathways. Conversely, enhancing TH17-cell-driven inflammation by treatment with prostaglandin analogues shifts the balance between *IL-12* and *IL-27* as compared with *IL-23* [41] and has adverse effects on the disease. Thus, there is good reason to suggest that therapies targeting the *IL-23/IL-17* pathway may provide a useful approach to the treatment of chronic inflammatory diseases. Elsewhere, we describe the development of a high throughput screen to test for agents with this ability [42]. It is noteworthy that several nutrients are able to affect this target, and we have previously shown that nutrition plays a very significant role in the development and symptomatology of the disease [43]. Thus, while much of the literature to date has focussed on the possibility of monoclonal antibodies or other pharmaceutical approaches, the possibility of nutritional intervention against this target should not be dismissed. Both pharmacogenomic and nutrigenomic approaches may be important in the control of disease in individuals carrying risk variants in *IL-12B* and/or *IL-23R* [3, 42, 43].

## Figures and Tables

**Table 1:** Summary of Crohn's disease and controls and Montreal classification data.

	Crohn's disease *n* (%)	Control group *n* (%)
Gender		
Female	223 (68.4)	199 (37.3)
Male	103 (31.6)	335 (62.7)

Age at first diagnosis		
Below 17	30 (10.5)	Not applicable
Between 17 and 40	204 (71.6)	
Above 40	51 (17.9)	

Crohn's disease location		
Colonic	86 (32.2)	Not applicable
Ileal	97 (36.3)	
Ileocolonic	84 (31.5)	

Crohn's disease behaviour		
Inflammatory	152 (57.4)	Not applicable
Penetrating	32 (12.1)	
Stricturing	81 (30.6)	
Ileal/stricturing	47 (40.2)	
Colonic/inflammatory	70 (59.8)	

Any other families and relatives with IBD		
Yes	88 (28.3)	Not applicable
No	223 (71.7)	

Bowel resection		
Yes	99 (31.8)	Not applicable
No	212 (68.2)	

Smoker at diagnosis/smoker at interview		
Yes	112 (46.7)	118 (29.4)
No	128 (53.3)	283 (70.6)

Extraintestinal manifestations		
Yes	44 (14.2)	Not applicable
No	267 (85.8)	

**Table 2:** Genotype and allele counts for* IL-12B* and *IL-23R* variants in CD patients and in Caucasian controls.

		Control *n* (%)	Case *n* (%)	OR (95% CI)	*P*
*IL-12B *	C/C	10 (2.9)	11 (3.6)	1.30 (0.54–3.12)	.638
rs1363670	C/G	71 (20.8)	70 (23.3)	1.17 (0.80–1.70)	
	G/G	260 (76.3)	220 (73.1)	1.00	
	HWE	0.10	0.08		
	Cochran-Armitage Trend for G	*Z* = −0.95, Pr < *Z*	0.172		
	C	91 (13.3)	92 (15.3)	1.17 (0.86–1.60)	.338
	G	591 (86.7)	510 (84.7)	1.00	

*IL-12B *	C/C	39 (10.8)	29 (9.2)	0.79 (0.47–1.35)	.625
rs6887695	C/G	177 (48.9)	148 (47.1)	0.89 (0.65–1.23)	
	G/G	146 (40.3)	137 (43.6)	1.00	
	HWE	0.21	0.25		
	C	255 (35.2)	206 (32.8)	0.90 (0.72–1.13)	.358
	G	469 (64.8)	422 (67.2)	1.00	

*IL-23R*	A/A	45 (12.5)	37 (11.8)	1.18 (0.72–1.93)	**.030**
rs10889677	A/C	146 (40.6)	158 (50.5)	1.55 (1.12–2.15)	
	C/C	169 (46.9)	118 (37.7)	1.00	
	HWE	0.15	0.18		
	A	236 (32.8)	232 (37.1)	1.21 (0.96–1.52)	.100
	C	484 (67.2)	394 (62.9)	1.00	

*IL-23R*	A/A	5 (0.8)	0 (0)	—	
rs11209026	A/G	75 (11.9)	28 (8.0)	0.64 (0.41–1.01)	.164
	G/G	553 (87.4)	321 (92.0)	1.00	
	HWE	0.19	1.00		
	Cochran-Armitage Trend for G	*Z* = 2.42, Pr > *Z*	0.008		
	A	85 (6.7)	28 (4.0)	0.58 (0.37–0.90)	**.015**
	G	1181 (93.3)	670 (96.0)	1.00	

*IL-23R*	C/C	173 (47.3)	123 (39.3)	1.00	.108
rs11805303	C/T	158 (43.2)	153 (48.9)	1.36 (0.99–1.88)	
	T/T	35 (9.6)	37 (11.8)	1.49 (0.89–2.49)	
	HWE	1.00	0.33		
	T	228 (31.1)	227 (36.3)	1.26 (1.01–1.58)	**.049**
	C	504 (68.9)	399 (63.7)	1.00	

*IL-23R*	A/A	56 (15.2)	16 (5.1)	0.29 (0.16–0.53)	**.0002**
rs1343151	A/G	154 (41.7)	142 (45.4)	0.95 (0.69–1.30)	
	G/G	159 (43.1)	155 (49.5)	1.00	
	HWE	0.07	0.02		
	Cochran-Armitage Trend for G	*Z* = 3.24, Pr > *Z*	0.0006		
	A	266 (36.0)	174 (27.8)	0.68 (0.54–0.86)	**.001**
	G	472 (64.0)	452 (72.2)	1.00	

*IL-23R*	G/G	72 (19.6)	29 (9.4)	0.42 (0.25–0.70)	**.001**
rs7517847	G/T	183 (49.7)	172 (55.7)	0.98 (0.70–1.38)	
	T/T	113 (30.7)	108 (34.9)	1.00	
	HWE	0.92	0.001		
	G	327 (44.4)	230 (37.2)	0.74 (0.59–0.92)	**.007**
	T	409 (55.6)	388 (62.8)	1.00	

**(a):** 

	*IL-12B* rs1363670		*IL-12B* rs6887695	
	OR (95% CI)	*P* value	OR (95% CI)	*P* value
Gender				
Female	1.54 (0.96–2.46)	0.086	0.79 (0.57–1.10)	.178
Male	0.85 (0.51–1.42)	0.608	0.93 (0.66–1.32)	.725

Age at first diagnosis				
0–16 years	1.41 (0.69–2.89)	0.317	0.76 (0.42–1.37)	.394
17–40 years	1.28 (0.90–1.81)	0.175	0.92 (0.71–1.19)	.513
>40 years	0.84 (0.43–1.63)	0.747	0.73 (0.46–1.15)	.184

CD location				
Colonic	1.16 (0.71–1.89)	0.523	0.94 (0.66–1.34)	.790
Ileal	1.39 (0.89–2.16)	0.153	0.55 (0.38–0.80)	**.002**
Ileocolonic	1.11 (0.68–1.80)	0.703	1.26 (0.89–1.79)	.206

CD behaviour				
Inflammatory	1.23 (0.84–1.81)	0.313	0.83 (0.62–1.11)	.220
Penetrating	0.46 (0.16–1.30)	0.161	1.06 (0.61–1.83)	.888
Stricturing	1.60 (1.01–2.53)	0.055	0.85 (0.59–1.23)	.408
Colonic/inflammatory	1.16 (0.69–1.96)	0.580	0.82 (0.56–1.21)	.332
Ileal/stricturing	1.86 (1.08–3.20)	**0.036**	0.58 (0.35–0.96)	**.035**
Any relatives with IBD	1.43 (0.91–2.25)	0.137	0.96 (0.68–1.36)	.859
Bowel resection	1.22 (0.77–1.92)	0.401	0.91 (0.65–1.28)	.609
Smoker at diagnosis	1.47 (0.79–2.74)	0.282	0.91 (0.60–1.39)	.668
Any EIMs	1.33 (0.73–2.42)	0.327	1.1 (0.70–1.74)	.724

**(b):** 

	*IL23R* rs10889677		*IL23R* rs11209026		*IL23R* rs11805303		*IL23R* rs1343151		*IL23R* rs7517847	
	OR (95% CI)	*P*	OR (95% CI)	*P*	OR (95% CI)	*P*	OR (95% CI)	*P*	OR (95% CI)	*P*
Gender										
Female	0.93 (0.67–1.29)	0.739	0.57 (0.31–1.05)	0.086	1.24 (0.89–1.73)	0.210	0.67 (0.48–0.94)	0.024*	0.79 (0.57–1.09)	.144
Male	0.75 (0.53–1.05)	0.113	0.51 (0.23–1.16)	0.119	1.32 (0.93–1.86)	0.129	0.67 (0.47–0.96)	0.028*	0.69 (0.49–0.97)	.041*
Age at first diagnosis										
0–16 years	0.60 (0.35–1.02)	0.064	0.24 (0.03–1.75)	0.175	1.56 (0.90–2.69)	0.110	0.41 (0.21–0.80)	0.007*	0.63 (0.36–1.10)	.105
17–40 years	0.81 (0.63–1.05)	0.116	0.61 (0.36–1.04)	0.073	1.32 (1.02–1.70)	**0.041**	0.62 (0.47–0.81)	0.0006*	0.65 (0.50–0.84)	.0008*
>40 years	0.76 (0.49–1.17)	0.216	0.73 (0.29–1.84)	0.675	1.34 (0.87–2.08)	0.205	0.84 (0.54–1.31)	0.506	0.91 (0.60–1.38)	.750

CD location										
Colonic	0.80 (0.57–1.13)	0.211	0.69 (0.33–1.45)	0.406	1.27 (0.90–1.80)	0.202	0.79 (0.55–1.13)	0.210	1.04 (0.74–1.45)	.864
Ileal	0.77 (0.55–1.07)	0.140	0.68 (0.34–1.38)	0.346	1.40 (1.00–1.95)	0.055	0.68 (0.48–0.97)	0.037*	0.62 (0.44–0.87)	.005*
Ileocolonic	0.76 (0.54–1.08)	0.123	0.34 (0.12–0.94)	**0.026**	1.38 (0.97–1.96)	0.080	0.43 (0.28–0.65)	0.00004*	0.52 (0.36–0.75)	.0003*

CD behaviour										
Inflammatory	0.78 (0.59–1.03)	0.096	0.73 (0.42–1.28)	0.296	1.31 (0.99–1.74)	0.067	0.69 (0.51–0.93)	0.013*	0.82 (0.62–1.08)	.165
Penetrating	0.76 (0.45–1.29)	0.333	0.23 (0.03–1.68)	0.179	1.40 (0.82–2.39)	0.255	0.38 (0.20–0.74)	0.003*	0.53 (0.30–0.92)	.025*
Stricturing	0.76 (0.53–1.09)	0.136	0.35 (0.13–0.97)	**0.037**	1.47 (1.03–2.10)	**0.040**	0.60 (0.40–0.89)	0.014*	0.56 (0.39–0.81)	.002*
Colonic/ inflammatory	0.80 (0.55–1.16)	0.243	0.63 (0.27–1.47)	0.363	1.26 (0.86–1.84)	0.274	0.76 (0.51–1.13)	0.206	1.05 (0.73–1.51)	.852
Ileal/stricturing	0.80 (0.51–1.26)	0.345	0.30 (0.07–1.24)	0.082	1.54 (0.98–2.41)	0.072	0.69 (0.42–1.13)	0.155	0.59 (0.37–0.94)	.032*
Any relatives with IBD	0.89 (0.63–1.26)	0.529	0.49 (0.21–1.14)	0.099	1.22 (0.86–1.73)	0.278	0.62 (0.43–0.90)	0.015*	0.64 (0.45–0.90)	.013*
Bowel resection	0.78 (0.56–1.08)	0.145	0.36 (0.14–0.90)	0.024*	1.33 (0.96–1.85)	0.101	0.61 (0.43–0.87)	0.008*	0.54 (0.38–0.76)	.0004*
Smoker at diagnosis	0.88 (0.57–1.37)	0.578	0.60 (0.28–1.29)	0.192	0.96 (0.62–1.49)	0.911	0.75 (0.48–1.16)	0.219	0.98 (0.65–1.48)	1.00
Any EIMs	0.64 (0.41–1.00)	0.057	0.32 (0.08–1.32)	0.116	1.46 (0.93–2.30)	0.116	0.52 (0.31–0.88)	0.016*	0.65 (0.41–1.03)	.069

*remains significant after multiple testing correction applied using the FDR.

**(a):** 

Haplotype	rs11805303	rs7517847	Hap-score	Control Hap-frequencies	Case hap-frequencies	Pool hap-frequencies	*P*	
1	C	G	−3.48	0.450	0.358	0.409	**.0005**	global-stat = 15.6
2	C	T	1.17	0.245	0.275	0.258	.241	df = 3
3	T	T	2.24	0.305	0.359	0.329	**.025**	**P** = .001
4	T	G	NA	3.4*E* − 10	0.008	0.003	NA	

**(b):** 

Haplotype	rs10889677	rs11209026	rs1343151	Hap-score	Control hap-frequencies	Case hap-frequencies	Pool hap-frequencies	*P*	
1	C	G	A	−2.69	0.296	0.232	0.268	**.007**	global-stat = 13.4
2	C	A	A	−1.90	0.069	0.044	0.058	.058	df = 4
3	C	G	G	1.62	0.310	0.350	0.328	.105	**P** = .009
4	A	G	G	1.91	0.323	0.374	0.346	.056	
5	A	G	A	NA	0.002	3.1*E* − 09	0.001	NA	
6	C	A	G	NA	6.1*E* − 10	NA	5.3*E* − 10	NA	

**Table 5:** The statistical interaction between *IL23R* SNPs and* NOD2* genotype.

	*NOD2*									
*IL23R*	d/d (*n* = 557)				d/D (*n* = 95)				D/D (*n* = 19)	
	Control	Case	OR (95% CI)	*P*	Control	Case	OR (95% CI)	*P*	Control	Case
rs10889677										
A/A	40	27	0.81 (0.46–1.40)		8	8	0.35 (0.11–1.15)	0.147	0	1
A/C	130	109	1.00	.103	12	34	1.00		2	8
C/C	156	88	0.67 (0.47–0.97)		14	19	0.48 (0.19–1.24)		0	8

rs11209026										
A/A	6	0	—		0	0	—		0	0
A/G	33	22	0.95 (0.54–1.68)	.986	5	2	0.20 (0.04–1.08)	0.061	0	1
G/G	289	202	1.00		29	59	1.00		2	16

rs11805303										
C/C	160	94	1.00		13	18	1.00	0.056	0	7
C/T	137	98	1.22 (0.85–1.75)	.140	12	37	2.23 (0.85–5.85)		2	9
T/T	28	29	1.76 (0.99–3.14)		8	6	0.54 (0.15–1.94)		0	1

rs1343151										
A/A	54	10	**0.23 (0.11–0.47)**		2	4	0.94 (0.16–5.69)	0.615	0	0
A/G	140	105	0.93 (0.65–1.33)	**.0003**	16	22	0.65 (0.27–1.55)		0	9
G/G	134	108	1.00		16	34	1.00		2	8

rs7517847										
G/G	64	24	**0.45 (0.26–0.79)**		4	3	0.60 (0.12–3.08)	0.156	0	0
G/T	172	121	0.84 (0.57–1.24)	**.018**	14	36	2.06 (0.84–5.07)		2	6
T/T	91	76	1.00		16	20	1.00		0	10

**Table 6:** Frequency of NOD2 genotypes in the study sample.

	Control *N* (%)	CD *N* (%)
rs2066844		
C/C	345 (94.8)	267 (86.7)
C/T	18 (5.0)	40 (13.0)
T/T	1 (0.3)	1 (0.3)

rs2066845		
C/C	0 (0)	0 (0)
C/G	3 (0.8)	16 (5.2)
G/G	361 (99.2)	292 (94.8)

rs2066847		
−/−	349 (95.9)	277 (89.9)
−/C	15 (4.1)	23 (7.5)
C/C	0 (0)	8 (2.6)
